# The TonB system in *Aeromonas hydrophila* NJ-35 is essential for MacA_2_B_2_ efflux pump-mediated macrolide resistance

**DOI:** 10.1186/s13567-021-00934-w

**Published:** 2021-04-29

**Authors:** Yuhao Dong, Qing Li, Jinzhu Geng, Qing Cao, Dan Zhao, Mingguo Jiang, Shougang Li, Chengping Lu, Yongjie Liu

**Affiliations:** 1 Joint International Research Laboratory of Animal Health and Food Safety, College of Veterinary Medicine, Nanjing Agricultural University, Nanjing, Jiangsu China; 2 Beijing Key Laboratory of Detection Technology for Animal-Derived Food Safety, College of Veterinary Medicine, China Agricultural University, Beijing, China; 3 Guangxi Key Laboratory for Polysaccharide Materials and Modifications, School of Marine Sciences and Biotechnology, Guangxi University for Nationalities, Nanning, China

**Keywords:** *Aeromonas hydrophila*, TonB system, Macrolides, MacA_2_B_2_ efflux pump, Sensitivity

## Abstract

The TonB system is generally considered as an energy transporting device for the absorption of nutrients. Our recent study showed that deletion of this system caused a significantly increased sensitivity of *Aeromonas hydrophila* to the macrolides erythromycin and roxithromycin, but had no effect on other classes of antibiotics. In this study, we found the sensitivity of *ΔtonB123* to all macrolides tested revealed a 8- to 16-fold increase compared with the wild-type (WT) strain, but this increase was not related with iron deprivation caused by *tonB123* deletion. Further study demonstrated that the deletion of *tonB123* did not damage the integrity of the bacterial membrane but did hinder the function of macrolide efflux. Compared with the WT strain, deletion of *macA*_*2*_*B*_*2*_, one of two ATP-binding cassette (ABC) types of the macrolide efflux pump, enhanced the sensitivity to the same levels as those of *ΔtonB123*. Interestingly, the deletion of *macA*_*2*_*B*_*2*_ in the *ΔtonB123* mutant did not cause further increase in sensitivity to macrolide resistance, indicating that the macrolide resistance afforded by the MacA_2_B_2_ pump was completely abrogated by *tonB123* deletion. In addition, *macA*_*2*_*B*_*2*_ expression was not altered in the *ΔtonB123* mutant, indicating that any influence of TonB on MacA_2_B_2_-mediated macrolide resistance was at the pump activity level. In conclusion, inactivation of the TonB system significantly compromises the resistance of *A. hydrophila* to macrolides, and the mechanism of action is related to the function of MacA_2_B_2_-mediated macrolide efflux.

**Supplementary Information:**

The online version contains supplementary material available at 10.1186/s13567-021-00934-w.

## Introduction

Gram-negative bacteria comprise most of the bacterial world. Different from Gram-positive bacteria, which only have a cytoplasmic membrane (CM), the cell envelope of Gram-negative bacteria contains two membranes, namely the CM and the outer membrane (OM) [1]. The OM protects Gram-negative bacteria from environmental hazards such as antibiotics and detergents [2]. Also, Gram-negative bacteria require rare essential nutrients such as iron and vitamins that are present in the extracellular environment at very low concentrations [3]. The solutes that have a molecular mass less than 600 Da can pass through OM porins by the concentration-gradient-driven passive diffusion [4]. However, the uptake of nutrients, especially those existing at extremely low concentrations, must occur by OM active transport. Due to the cell envelope architecture, there is no electrochemical gradient powering the active transport across the OM and no ATP in the periplasmic space, which means that OM transporters need to extract energy from the CM. The energy transfer is commonly carried out by trans-periplasmic proteins, for example, the CM-anchored TonB complex [5]. TonB system, consisting of TonB, ExbB and ExbD proteins, couples the proton motive force (PMF) of the CM to energize active transport across the OM by the TonB-dependent transporters (TBDTs) [6]. TonB protein, the main component of this system, is CM-anchored by a transmembrane helix, and stabilized by ExbB and ExbD [7]. TonB system was thought to be only involved in nutrient import. In *Escherichia coli*, TonB system provides energy to the OM protein BtuB for vitamin B_12_ transport [3]. In *Vibrio anguillarum*, TonB1 system is responsible for heme and ferrichrome transport, while TonB2 system is essential for the transport of endogenous and exogenous siderophores [8]. However, it is now becoming clear that the uptake of nutrient is only one of the many potential functions of the TonB system. Indeed, in *Myxococcus xanthus*, TonB system is required for the secretion of the protease PopC, which suggests that TonB system not just participates in import processes, but also is involved in the secretion of intracellular proteins [9].

*Aeromonas hydrophila* is one of the most important fish pathogens causing haemorrhagic septicaemia. Also, this bacterium is associated with a variety of human illnesses, such as sepsis, wound infections, and food-borne gastroenteritis [10]. To date, antibiotics are still the most effective way to fight this bacterial infection. However, the widespread use of antibiotics has led to the emergence of antimicrobial resistance in *A. hydrophila* and bioaccumulation in host tissues, which seriously threatened human and public health. Some strains of *A. hydrophila* have been found to be resistant to macrolides, tetracyclines, sulfonamides, and quinolones [11, 12]. In response to this phenomenon, understanding of resistance mechanisms has become an urgent necessity for development of an effective therapeutic strategy against this pathogen.

Recently, three *tonB* genes in *A. hydrophila* NJ-35, namely *tonB1*, *tonB2* and *tonB3*, were described to be involved in antibiotic resistance. A triple-deletion mutant of *tonB123* showed a significantly increased sensitivity to the macrolide antibiotics erythromycin and roxithromycin, but had no effect on other classes of antibiotics [13]. In this study, we further demonstrated that the increased susceptibility of *ΔtonB123* mutant to macrolides was due to the decrease in drug efflux, and furthermore, associated with the MacA_2_B_2_-mediated pump.

## Materials and methods

### Bacterial strains and growth conditions

The bacterial strains and plasmids used in this study are listed in Additional file 1. *A. hydrophila* NJ-35 (accession number CP006870), which belongs to the ST251 clonal group, was isolated from dead cultured cyprinoid fish in the Jiangsu province of China in 2010.

*A. hydrophila* and *E. coli* were routinely grown in Luria–Bertani (LB) medium at 28 °C and 37 °C, respectively. When necessary, the medium was supplemented with the following antibiotics: chloramphenicol (Cm), 34 mg/L for *E. coli*; ampicillin (Amp), 100 mg/L for *A. hydrophila.* All reagents used in this study were supplied by Sigma (St. Louis, MO, USA) unless otherwise indicated.

### Minimum inhibitory concentration (MIC) assay

*A. hydrophila* NJ-35 and its derivative *tonB123* mutant grown to logarithmic phase were assayed for macrolide sensitivity. MICs were determined by broth microdilution, following the Clinical and Laboratory Standards Institute (CLSI) guidelines [14]. Briefly, cultured cells in the log phase were diluted to 2 × 10^5^ cells/mL in fresh Mueller–Hinton broth (MHB). The inoculum (100 μL) was added to each well of 96-well plates. Antibiotic was then added to the first wells and twofold dilutions were performed. Plates were incubated at 28 °C for 18 h. The MIC value was measured three times and the average of the measured values was determined as the MIC value for the strain and the antibiotic. Macrolide antibiotics, including roxithromycin (ROX), erythromycin (ERY), tilmicosin (TIL), tylosin (TYL), acetylspiramycin (ACE), azithromycin (AZI), dirithromycin (DIR) and medemycin (MED) were purchased from Solarbio (Beijing, China).

To determine whether iron was involved in the effect on the bacterial sensitivity to macrolides, MIC of each macrolide was examined in MHB supplemented with 36 μM FeCl_3_ or 150 μM 2, 2-dipyridyl (DIP).

### Cell membrane integrity

To determine whether deletion of *tonB123* resulted in damage to the permeability barrier of the bacterial cell membrane, cell membrane integrity was examined by determining the release of cytoplasmic constituents into the supernatant, such as nucleic acids and proteins [15]. The bacteria were incubated at 28 °C for 6 h, and then immediately centrifuged (9000 *g*) for 5 min at 4 °C. The supernatant was filter-sterilized using 0.22-µm (pore-size) membrane. The amount of nucleic acids released from the cytoplasm was determined by measuring the optical density at 260 nm. The concentration of proteins in the supernatant was determined using Protein Bradford Assay kit (Thermo Fisher Scientific, Waltham, USA).

### Morphological observation

Bacterial cell morphology was evaluated using scanning electron microscope (SEM) and transmission electron microscope (TEM) [16]. For SEM examination, the dehydrated samples were treated thrice with 100% tert-butanol and dried with a freeze dryer for 2 h. The samples were placed on stubs and coated with gold film by sputter coating and viewed using a FEI Quanta FEG250 scanning electron microscope. For TEM examination, the dehydrated samples were infiltrated, embedded in araldite and processed to a trapezoid shape having a surface area of less than 0.2 mm × 0.2 mm. Ultramicrotomy was performed on the embedded material to obtain a thickness of 50–90 nm. Subsequently, the thin sections were mounted onto 300 mesh copper grids, stained with alcoholic uranyl acetate and alkaline lead citrate, and washed with distilled water. The samples were observed on a Hitachi 600 transmission electron microscope.

### Drug accumulation assay

To determine the influence of TonB system on macrolide efflux, the intracellular drug accumulation was examined with the WT and *ΔtonB123* strains. A single colony of each strain was cultured in LB medium at 28 °C for 6 h, after which the cells were transferred to 100 mL LB medium and cultured to the logarithmic-phase (OD_600_ = 0.6). Cells were pelleted by centrifugation at 4000 *g* for 10 min, and resuspended in PBS to a turbidity at 600 nm of about 3.0. The cells were then incubated for 15 min at 28 °C, and tilmicosin was added to reach the final concentration of 10 mg/L. 1 mL aliquots of culture were removed from the tube every 5 min. Then cells were harvested by centrifugation at 800 *g* for 10 min. The pellet was washed three times with PBS, followed by sonication on ice, then centrifuged at 10 000 *g* for 10 min to remove cellular debris. The intracellular concentration of tilmicosin was analysed by high-performance liquid chromatography coupled with tandem mass spectrometry (HPLC–MS/MS) [17].

### Effect of efflux pump inhibitors on the sensitivity of *A. hydrophila* to macrolides

To assess the contribution of the efflux pump to TonB-dependent macrolide efflux, MIC levels were determined using broth microdilution assay in the absence or presence of 3 mg/L carbonyl cyanide m-chlorophenylhydrazone (CCCP), 50 mg/L phenylalanine-arginine β-naphthylamide (PAβN) or 10 mg/L sodium orthovanadate (SOV). The final concentrations of the pump inhibitors were selected based on a preliminary sighting study, in which the highest concentration of efflux pump inhibitor that does not affect bacterial growth was determined.

### Inactivation of the target gene

Gene mutants were constructed by homologous recombination using the suicide plasmid pYAK1 as previously described [13]. The primer pairs are shown in Additional file 2. Briefly, the upstream and downstream flanking regions of the target gene were PCR amplified from the chromosomal DNA of *A. hydrophila* NJ-35, and then ligated in-frame using fusion PCR. The fusion fragment was cloned into the pYAK1 suicide plasmid and then chemically transformed into *E. coli* SM10 competent cells. The donor strain *E. coli* SM10-pYAK1 and the recipient strain *A. hydrophila* were mixed at a ratio of 2:1 (vol/vol) in medium, spotted on a nylon filter on an LB plate and conjugated for 12 h at 28 °C. The bacteria were washed from the filter and grown on LB plates containing Amp and Cm. The positive colonies were verified by PCR and then inoculated in LB broth supplemented with 20% sucrose to induce a second crossover event. The suspected mutants were verified by PCR. Using the same approach, additional deletion mutants were also constructed.

The corresponding complemented strains of the mutants were constructed with pMMB207. The target gene was isolated by PCR amplification and then ligated into pMMB207. The verified complementation vector was transferred into *E. coli* SM10 by chemical transformation and then transformed into the mutant strain by conjugation. The transconjugants were selected on LB agar containing Amp and Cm and further confirmed by PCR.

### Quantitative reverse transcription PCR (qRT-PCR)

Total RNA was extracted from cultures in the exponential growth phase with an E.Z.N.A. bacterial RNA isolation kit (Omega, USA). The RNA was reverse transcribed to cDNA using HiScript II QRT Supermix (Vazyme, China). The mRNA transcription levels were examined using a One Step qRT-PCR SYBR Green kit (Vazyme Biotech) in an ABI PRISM 7300 Fast Real-time PCR machine. All qRT-PCR operations were performed in triplicate. The housekeeping gene *recA* was chosen as an internal control for qRT-PCR, and the fold-change of mRNA expression levels was calculated according to the 2^−∆∆CT^ method [18]. The primer pairs are shown in Additional file 2.

### Statistical analyses

Data were analysed using SPSS16.0 software (SPSS Inc., Chicago, IL, USA). Multiple comparisons were performed using a Student *t* test and analysis of variance (ANOVA) followed by Bonferroni’s post hoc-test. *P*-values < 0.05 were considered to be statistically significant.

## Results

### Inactivation of *tonB123* enhances *A. hydrophila* sensitivity to macrolide antibiotics

To confirm the role of TonB system in macrolide sensitivity of *A. hydrophila*, we tested the sensitivity of the WT and *ΔtonB123* strains to common macrolides, including erythromycin, roxithromycin, dirithromycin, azithromycin, acetylspiramycin, tilmicosin, tylosin and medemycin. The MIC data showed that the sensitivity of *ΔtonB123* strain to all above macrolides increased 8- to 16-fold compared with the WT strain (Table 1). This defect could be partially complemented by any of the three *tonB* genes. These results indicated an important role of TonB system in macrolide resistance.

**Table 1:** **Influence of *tonB123* deletion on the susceptibility of** ***A. hydrophila* strains to macrolide**

Strain	MIC (mg/L)							
	ROX	ERY	TIL	TYL	ACE	AZI	DIR	MED
WT	32	16	64	128	256	1	128	128
Δ*tonB123*	2	1	4	16	16	0.125	16	8
*ΔtonB123* + *ptonB1*	16	8	32	64	64	1	64	128
*ΔtonB123* + *ptonB2*	16	8	32	64	64	1	64	128
*ΔtonB123* + *ptonB3*	8	4	16	32	32	0.25	64	64
Δ*macA*_*1*_*B*_*1*_	32	16	64	128	256	1	128	128
Δ*tonB123*Δ*macA*_*1*_*B*_*1*_	2	1	4	16	16	0.125	16	8
Δ*macA*_*2*_*B*_*2*_	2	1	4	16	16	0.125	16	8
Δ*tonB123*Δ*macA*_*2*_*B*_*2*_	2	1	4	16	16	0.125	16	8
Δ*macA*_*2*_*B*_*2*_ + *pmacA*_*2*_*B*_*2*_	32	16	64	128	256	1	128	128
Δ*tonB123*Δ*macA*_*2*_*B*_*2*_ + *pmacA*_*2*_*B*_*2*_	2	1	4	16	16	0.125	16	8

ROX: roxithromycin, ERY: erythromycin, TIL: tilmicosin, TYL: tylosin, ACE: acetylspiramycin, AZI: azithromycin, DIR: dirithromycin, MED: medemycin.

### Inactivation of *tonB123* does not damage the bacterial membrane

**Figure 1:**
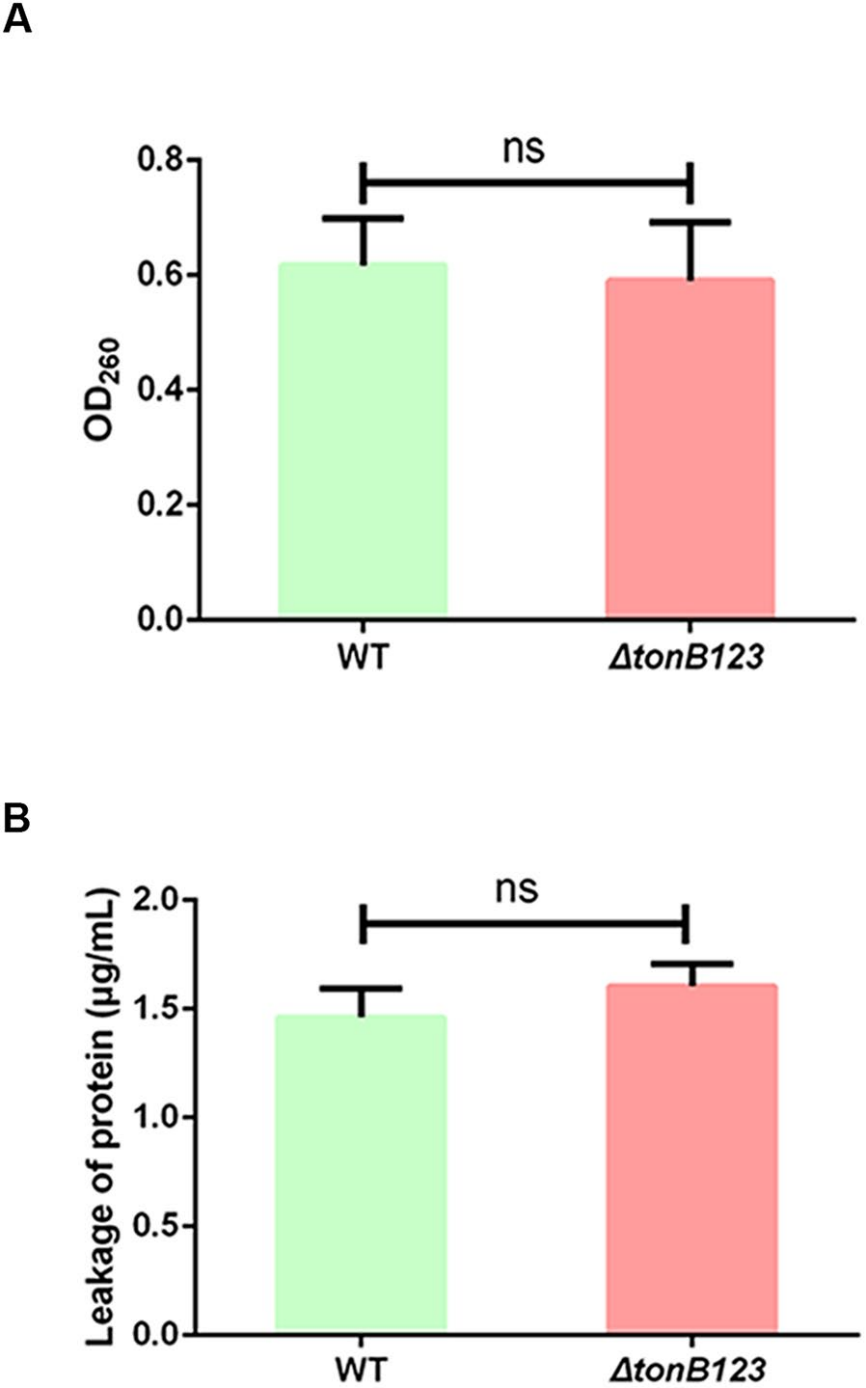
**Leakage of nucleic acids (A) and proteins (B) from the WT and ΔtonB123 mutant.** Nucleic acids leakage was assessed by measuring the absorbance of the supernatants of bacterial cultures at OD_260_. The protein concentration in supernatants was determined by the Bradford’s method. Data represent the means ± SEM from three independent experiments. “ns” signifies not statistically significant.

**Figure 2:**
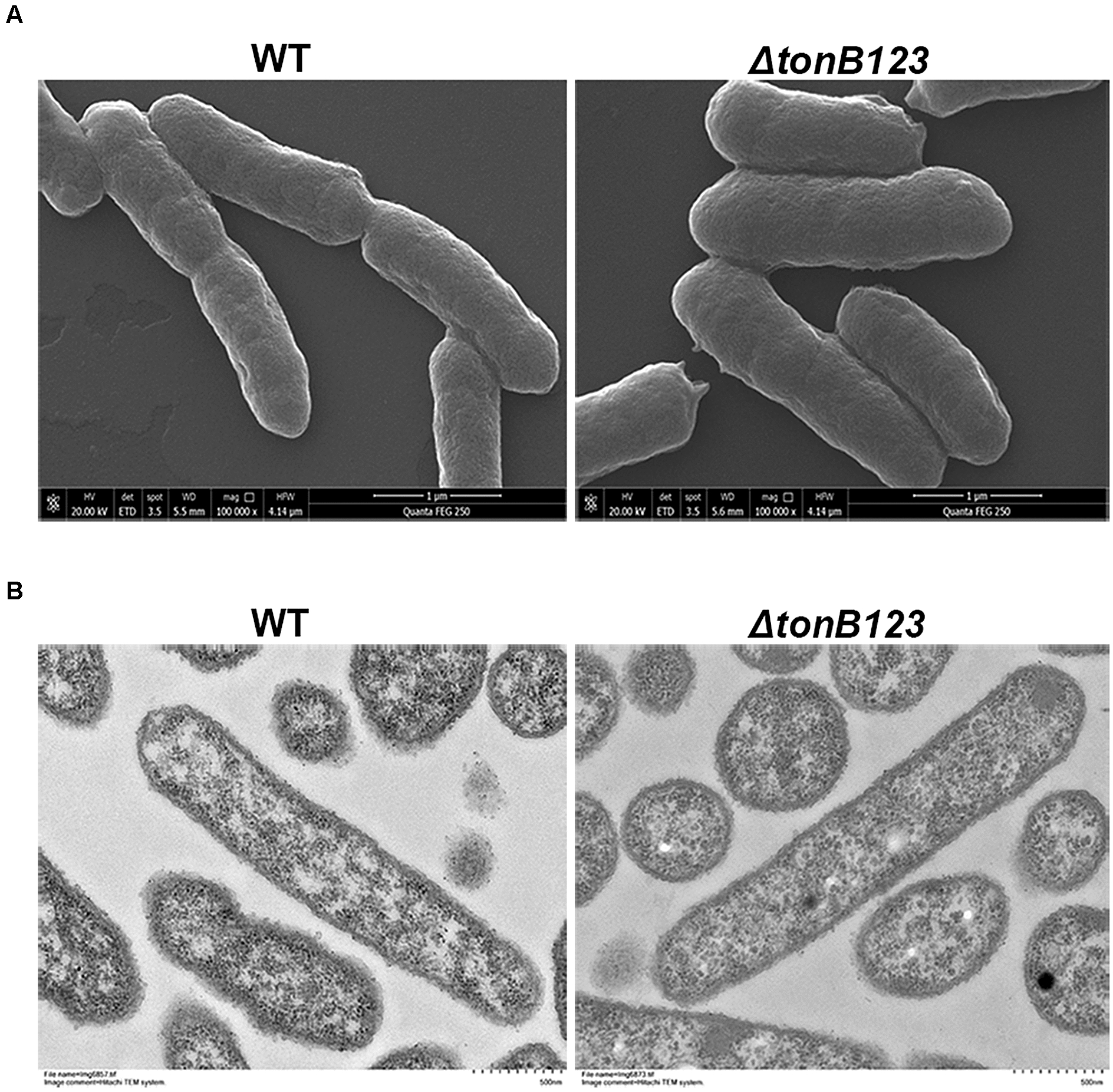
**Bacterial cell morphology of the WT and ΔtonB123 mutant.** **A** SEM images of the WT and *ΔtonB123* mutant. Scale bar = 1 μm. **B** TEM images of the WT and *ΔtonB123* mutant. Scale bar = 500 nm. At least three random fields were observed and analyzed, from three independent experiments.

To determine whether inactivation of the TonB system disrupted the cell membrane integrity, we monitored the leakage of cell constituents through the bacterial membrane. As shown in Figure 1A, the amount of nucleic acids released in *ΔtonB123* was not different from that of the WT strain. Similarly, the WT and *ΔtonB123* strains had the same amount of protein leakage (Figure 1B). In addition, as demonstrated by SEM and TEM, both the WT and *ΔtonB123* strains displayed intact cell membrane and smooth surface, with no observable difference (Figure 2).

### Iron availability does not influence the sensitivity of *A. hydrophila* to macrolides

**Figure 3:**
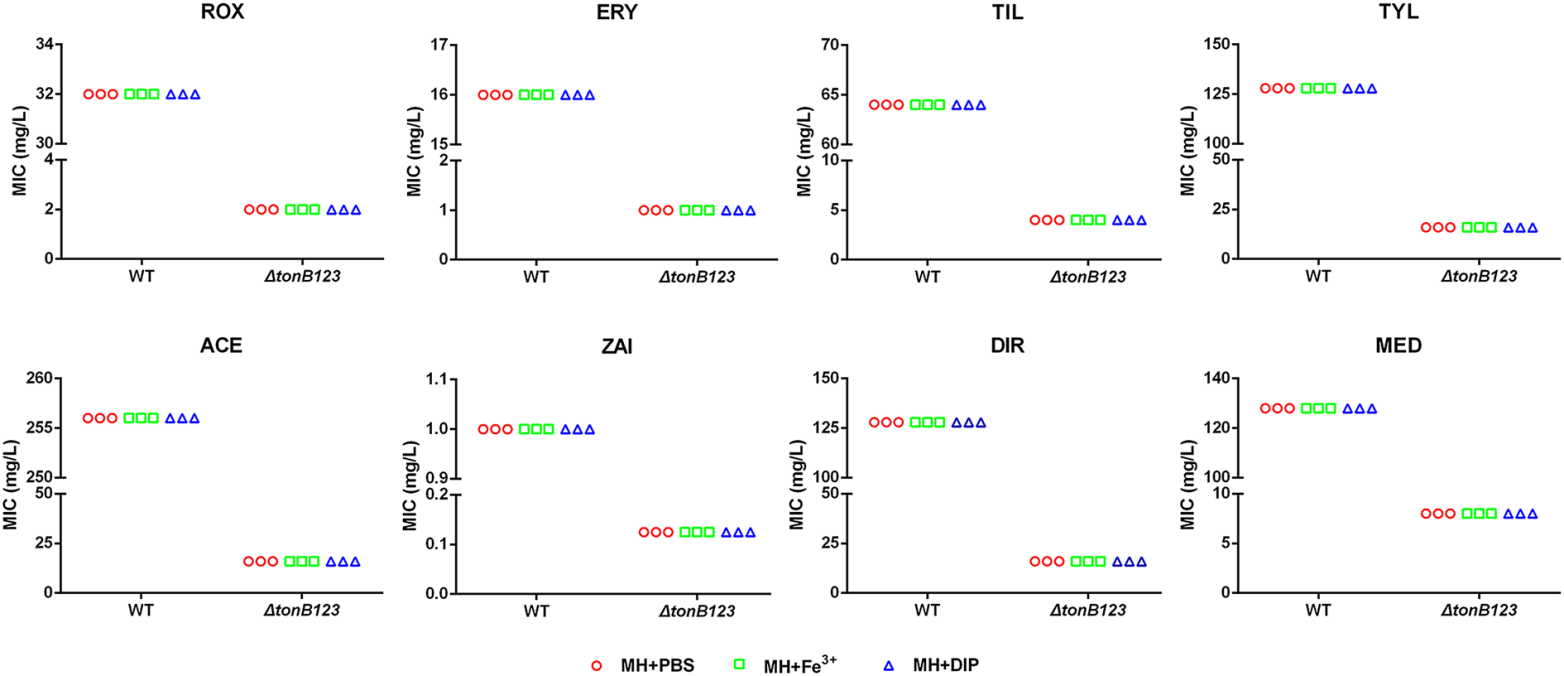
**Influence of iron on macrolide resistance of the WT and ΔtonB123 mutant.** The MICs were determined in MH broth containing 36 μM FeCl_3_ (iron-supplemented) or 150 μM DIP (iron-limited). The data represent three independent biological replicates.

To investigate if iron deprivation could influence macrolide susceptibility of *A. hydrophila*, we determined the MIC of each macrolide in MH broth supplemented with an excess of iron (36 μM FeCl_3_). Our data revealed that the sensitivity of *ΔtonB123* mutant to macrolides increased by 8- to 16-fold compared to that of the WT strain, regardless of iron presence (Figure 3). The results suggested that iron surplus could not restore the macrolide susceptibility in the *ΔtonB123* mutant*.* To confirm this finding, we determined the MIC of each macrolide in the presence of the iron chelator DIP. The results showed that iron deprivation also had no effect on the macrolide sensitivity of the WT or *ΔtonB123* strains (Figure 3).

### Inactivation of *tonB123* hinders macrolide efflux function

**Figure 4:**
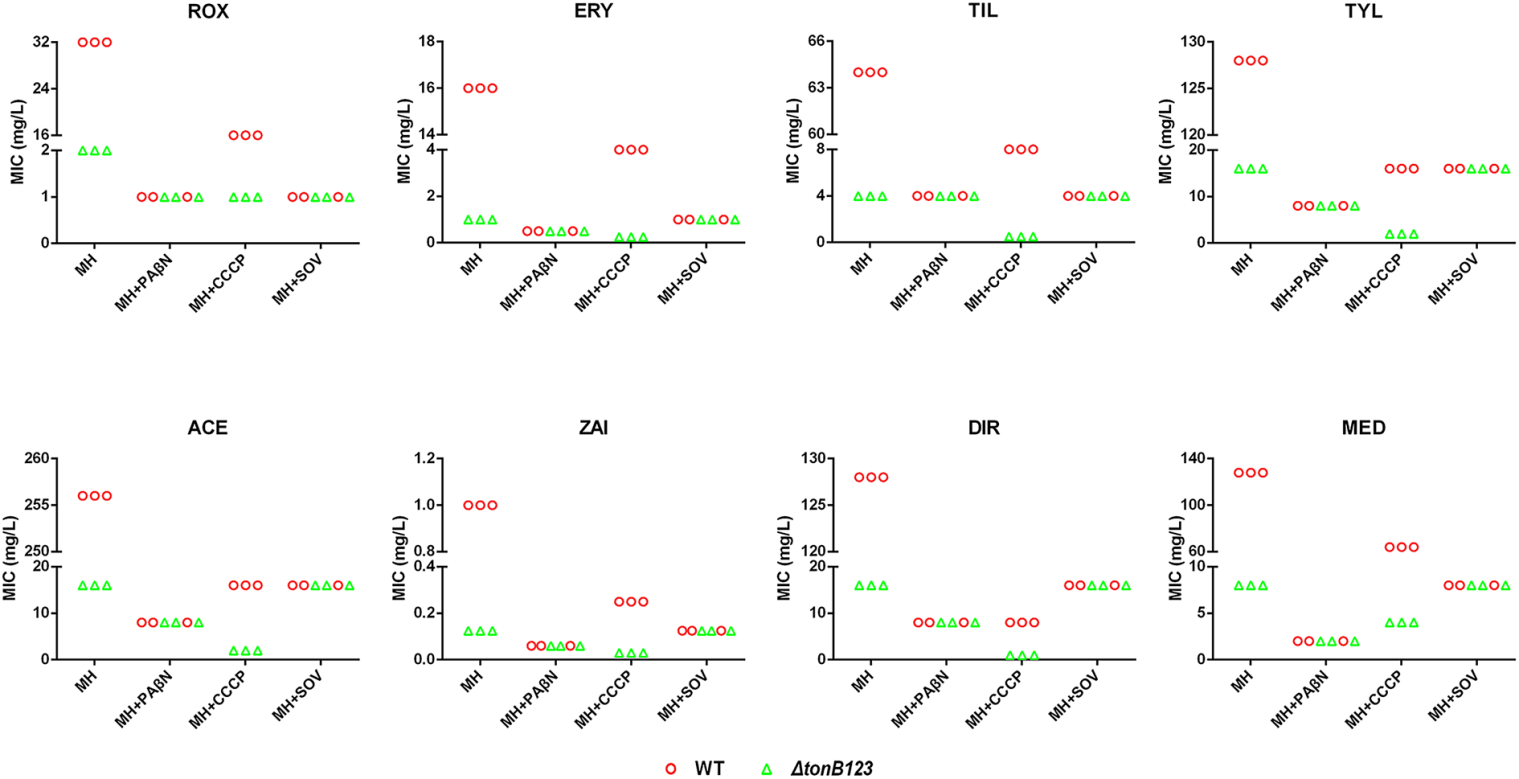
**Effect of efflux pump on TonB-dependent macrolide efflux in the WT and ΔtonB123 mutant.** The MICs were determined in the absence or presence of 3 mg/L CCCP, 50 mg/L PAβN or 10 mg/L SOV. The data represent three independent biological replicates.

**Figure 5:**
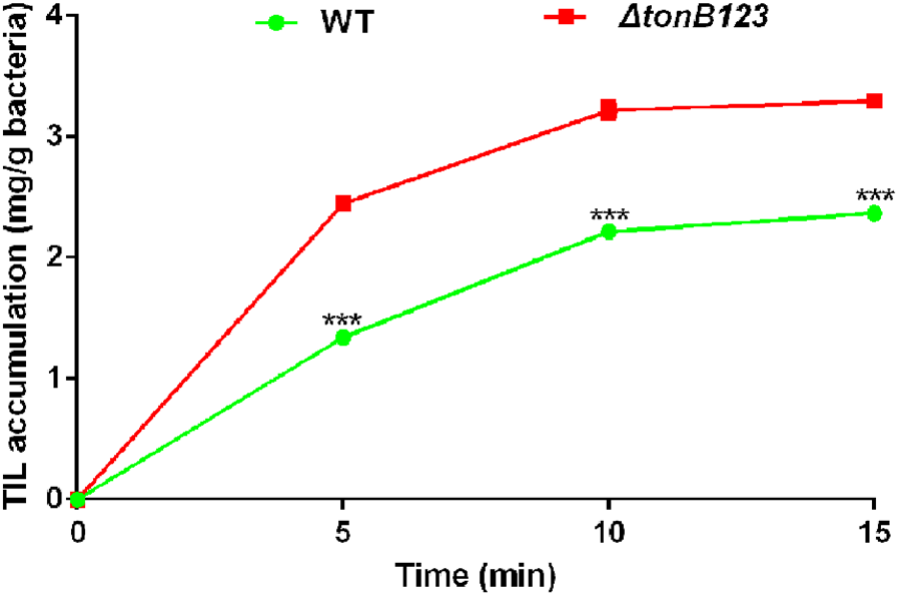
**Accumulation of tilmicosin in the WT and ΔtonB123 mutant.** The intracellular concentration of tilmicosin was analysed by HPLC–MS/MS. Data represent the means ± SEM from three independent experiments. ***P < 0.001.

To investigate whether the effect of TonB system on macrolide resistance is related to the efflux pump activity, we determined the MIC of macrolides in presence of the efflux pump inhibitor PAβN. Our data showed that PAβN eliminated the difference of macrolide resistance between the WT strain and *ΔtonB123* mutant (Figure 4). This finding suggests that efflux pumps might be involved in mediating macrolide resistance in the *ΔtonB123* mutant. To further assess whether the increased sensitivity of *ΔtonB123* results from the disruption in function of antibiotic efflux, a tilmicosin accumulation assay was performed with the WT and *ΔtonB123* strains*.* As demonstrated in Figure 5, the *ΔtonB123* mutant accumulated more tilmicosin compared to the WT strain (*P* < 0.001). These data indicate that deletion of TonB system might somehow influence the behaviour of *A. hydrophila* in macrolide efflux.

### ATP-binding cassette (ABC) family efflux is involved in TonB-dependent macrolide resistance

To explore which kind of pump was involved in TonB-dependent macrolide efflux, we determined the MICs of macrolides against WT and *ΔtonB123* strains in the presence of CCCP, an ionophore that disrupts PMF. The results showed that addition of CCCP caused significantly increased susceptibility of both the WT and *ΔtonB123* strains to all macrolides, but a significant difference in macrolide sensitivity between the two strains was still present (Figure 4). However, when the ABC family efflux was restrained by the ATPase inhibitor SOV, the difference in sensitivity to macrolides between WT and *ΔtonB123* disappeared completely (Figure 4). These data suggested that the TonB system might impact ABC family pump-mediated macrolide efflux.

### ***TonB is essential for the function of the MacA***_***2***_***B***_***2***_*** efflux pump***

The macrolide-specific ABC-type efflux pump MacAB has been identified in diverse Gram-negative bacteria [19–21]. In the genome of *A. hydrophila* NJ-35, two putative open reading frame (ORF) clusters, MacA_1_B_1_ and MacA_2_B_2_, were retrieved. To determine whether the effect of the TonB system on macrolide resistance was mediated by these two pumps, we deleted the *macA*_*1*_*B*_*1*_ and *macA*_*2*_*B*_*2*_ loci, respectively, in the WT and *ΔtonB123* background. As shown in Table 1, inactivation of *macA*_*1*_*B*_*1*_ had no effect on macrolide susceptibility of the two strains, but deletion of *macA*_*2*_*B*_*2*_ in the WT strain enhanced the susceptibility to the same levels as those of the *ΔtonB123* mutant. Notably, deletion of *macA*_*2*_*B*_*2*_ in the *ΔtonB123* mutant did not further enhance the improved bacterial susceptibility to macrolides. This finding indicated that macrolide resistance afforded by the MacA_2_B_2_ pump was completely abrogated by deletion of *tonB123*.

**Figure 6:**
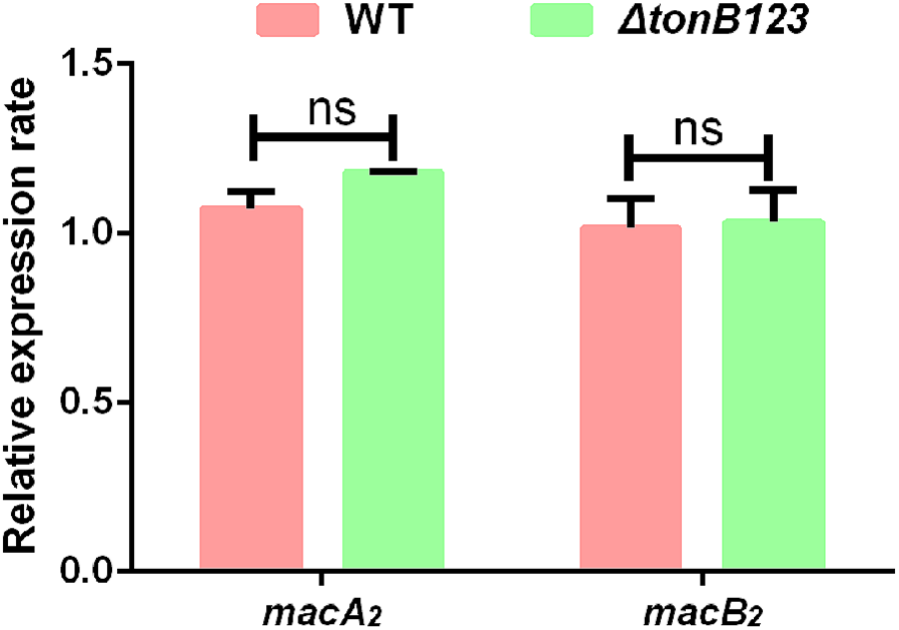
**The mRNA expression levels of two components of the macA**_***2***_***B***_***2***_** locus in the WT and ΔtonB123 mutant.** RNA was isolated from the log-phase bacteria that had been cultured in LB broth. The *recA* gene was chosen as the internal control. The results are expressed as n-fold increase with respect to the control. Data represent the means ± SEM from three independent experiments. “ns” signifies not statistically significant.

Further, we evaluated the mRNA transcription levels of two components of the *macA*_*2*_*B*_*2*_ locus in the WT and *ΔtonB123* strains by qRT-PCR. No significant difference was observed between the two strains (Figure 6), indicating that the effect of the TonB system on MacA_2_B_2_-mediated macrolide resistance was not related to the gene expression level of the efflux pump.

## Discussion

In agreement with our recent investigations, this study further demonstrated the involvement of the TonB system in macrolide resistance of *A. hydrophila*. The TonB system in *Pseudomonas aeruginosa* has been reported to be related to resistance to various classes of antibiotics, such as quinolones, macrolides, penicillins, and β-lactams [22]. In *Neisseria gonorrhoeae*, the TonB system was required for antimicrobial hydrophobic agents, as *tonB-exbB-exbD* deletion mutant showed increased sensitivity to erythromycin, polymyxin B and detergent Triton X-100 [23]. Different from the above two reports, our previous findings for *A. hydrophila* demonstrated that the TonB system was only involved in tolerance to macrolides.

The TonB system is an energy transduction complex that consists of the ExbB, ExbD and TonB proteins which delivers energy from the CM to the OM for nutrient transport [24]. Our previous study indicated that the TonB system in *A. hydrophila* NJ-35 was involved in iron transport [13]. It is well known that intracellular iron homeostasis plays an important role in bacterial antibiotic resistance. Iron deprivation is known to be able to impair the activity of drug efflux pump in *Mycobacterium tuberculosis* [25]. In this study, however, addition of an iron overload did not change the sensitivity of the wild-type *A. hydrophila* or its *tonB123* mutant. This finding indicates that the enhanced effect of *tonB123* deletion on macrolides susceptibility in *A. hydrophila* was independent of its influence on iron availability.

There is a great variety of resistance mechanisms observed in bacteria. Of them, the OM barrier and efflux pumps are the two most common resistance mechanisms [26]. The former limits the rate of antibiotic uptake into the cell, while the latter can expel drug into the external surrounding environment [22]. The TonB system has been demonstrated to be necessary for maintaining membrane integrity in *Pseudomonas putida*, and its deficiency leads to increased membrane permeability [27]. In this study, however, inactivation of this system did not affect membrane permeability. Moreover, increased sensitivity observed in the *ΔtonB123* was shown to be drug specific. We also tested whether functioning of the efflux pump for macrolide extrusion was compromised in the *ΔtonB123* mutant. The difference between the WT and *ΔtonB123* strains in macrolide sensitivity disappeared in the presence of a broad-spectrum efflux pump inhibitor PAβN, suggesting that the TonB system is associated with efflux of macrolides. Bacterial efflux systems generally fall into five classes, the major facilitator (MF) superfamily, the resistance-nodulation-division (RND) family, the small multidrug resistance (SMR) family, the multidrug and toxic compound extrusion (MATE) family and the ABC family [28]. Except for the ABC transporters, which use the ATP as an energy source to transport the drugs across the membrane, the others described are PMF-dependent multidrug efflux pumps [29]. Several previous studies have shown that the TonB system participates in the efflux of antibiotics through RND efflux pumps, for example, MexAB-OprM pump in *P. aeruginosa* [22] and MtrC-MtrD-MtrE pump in *N. gonorrhoeae* [23]. However, our study shows for the first time that the effect of the TonB system in *A. hydrophila* macrolide resistance was mediated by MacA_2_B_2_ efflux pump, which has been reported as an ABC family pump.

In Gram-negative bacteria, MacAB efflux pumps usually cooperate with outer membrane channel TolC to fulfill the efflux function [30]. MacAB forms a tripartite channel with TolC to drive the efflux of macrolides out of the bacterial cells [31]. Given that the function of the TonB system is to energize the TBDTs in the OM for the transport of nutrients, it is tempting to speculate that TonB proteins may transfer PMF from the cytoplasmic membrane to TolC to allow for antibiotic efflux. In this study, however, the protein domain predicted used I-TASSER online server [32] showed that TolC did not contain a beta-barrel or lumen-occluding cork domain with an essential sequence called the TonB box (Additional file 3), which was the typical structure of TBDT BtuB (Protein Data Bank accession number 1NQH) [33, 34]. Therefore, TolC is unlikely to be a TonB-dependent gated channel. A common feature in action mechanisms of TonB system is that TonB behaves as a regulating protein that influences the conformation of outer membrane proteins. Therefore, we speculate that TonB may be indirectly involved in the allosteric mechanism of TolC by protein–protein interactions. However, we cannot rule out the possibility that TonB deficiency may interfere with the activation of the pump components located in the cytoplasmic membrane, making the pump nonfunctional. Nevertheless, these questions are actively being studied in our group and remain exciting for the future.

Taken together with our recent report, these results support the notion that TonB system plays an important role in macrolide resistance of *A. hydrophila*. Although the exact mechanism warrants continued study, the TonB system is clearly involved in the resistance action by inhibiting the function of MacA_2_B_2_-mediated macrolide efflux. Further elucidation of the mechanism of action will undoubtedly contribute to the development of new antimicrobial agents.

## Supplementary Information

**Additional file 1.** **Bacterial strains and plasmids used in this study.**

**Additional file 2.** **Primers used in this study.**

**Additional file 3.** **The 3D structure of TolC (A) and BtuB (B).** The 3D structure of TolC (U876_06560) was predicted used I-TASSER online server. Three TolC monomers assemble to form a continuous conduit containing a 12-stranded β-barrel and a α-helical barrel. The monomers are individually coloured. In the single protomer, the β-barrel domain is in blue, the a-helical barrel domain is in orange. BtuB is the *E. coli* TonB-dependent vitamin B_12_ transporter. In BtuB, TonB box is in red, luminal domain is in purple and the β-barrel is in yellow. Protein Data Bank accession number 1NQH.

## Data Availability

All data generated or analysed during this study are included in this published article and its Additional files.

## Abbreviations

WT: Wild-type
ABC: ATP-binding cassette
CM: Cytoplasmic membrane
OM: Outer membrane
PMF: Proton motive force
TBDTs: TonB-dependent transporters
LB: Luria–Bertani
Cm: Chloramphenicol
Amp: Ampicillin
MIC: Minimum inhibitory concentration
CLSI: Clinical and Laboratory Standards Institute
MHB: Mueller Hinton broth
DIP: 2,2-Dipyridyl
SEM: Scanning electron microscope
TEM: Transmission electron microscope
OD: Optical density
HPLC–MS/MS: High-performance liquid chromatography coupled with tandem mass spectrometry
CCCP: Carbonyl cyanide m-chlorophenylhydrazone
PAβN: Phenylalanine-arginine β-naphthylamide
SOV: Sodium orthovanadate
PCR: Polymerase chain reaction
ORF: Open reading frame
MF: Major facilitator
RND: Resistance-nodulation-division
SMR: Small multidrug resistance
MATE: Multidrug and toxic compound extrusion
ATP: Adenosine triphosphate
ROX: Roxithromycin
ERY: Erythromycin
TIL: Tilmicosin
TYL: Tylosin
ACE: Acetylspiramycin
AZI: Azithromycin
DIR: Dirithromycin
MED: Medemycin
