# Supplementary material for: The TonB system in Aeromonas hydrophila NJ-35 is essential for MacA2B2 efflux pump-mediated macrolide resistance
Source: Vet Res. 2021 Apr 29;52:63. doi: 10.1186/s13567-021-00934-w (PMC8082627; doi:10.1186/s13567-021-00934-w)
Supplement: Supplementary file 1 — Additional file 1. Bacterial strains and plasmids used in this study. [file 13567_2021_934_MOESM1_ESM.doc]

**Additional file 1 Bacterial strains and plasmids used in this study**

| **Strain or plasmid** | **Description** | **Source** |
| --- | --- | --- |
| **Strain** |  |  |
| NJ-35 | Wilde-type, isolated from diseased crucian carp, in China | Laboratory collection |
| *ΔtonB123* | A triple-deletion mutant of *tonB1*, *tonB2* and *tonB3* from NJ-35 | Laboratory collection |
| *ΔtonB123+ptonB1* | Δ*tonB123* complemented with pMMB-*tonB1* | Laboratory collection |
| *ΔtonB123+ptonB2* | Δ*tonB123* complemented with pMMB-*tonB2* | Laboratory collection |
| *ΔtonB123+ptonB3* | Δ*tonB123* complemented with pMMB-*tonB3* | Laboratory collection |
| *ΔmacA_1_B_1_* | *macA_1_B_1_* deletion mutant from NJ-35 | This study |
| *ΔtonB123ΔmacA_1_B_1_* | *macA_1_B_1_* deletion mutant from *ΔtonB123* | This study |
| *ΔmacA_2_B_2_* | *macA_2_B_2_* deletion mutant from NJ-35 | This study |
| *ΔtonB123ΔmacA_2_B_2_* | *macA_2_B_2_* deletion mutant from *ΔtonB123* | This study |
| *ΔmacA_2_B_2_+pmacA_2_B_2_* | *ΔmacA_2_B_2_* complemented with pMMB-*macA_2_B_2_* | This study |
| *ΔtonB123ΔmacA_2_B_2_+pmacA_2_B_2_* | *ΔtonB123ΔmacA_2_B_2_* complemented with pMMB-*macA_2_B_2_* | This study |
| **Plasmid** |  |  |
| pYAK1 | R6K-ori suicide vector, SacB^+^, Cm | Laboratory collection |
| pYAK1-*macA_1_B_1_* | pYAK1 carrying the flanking sequence of *macA_1_B_1_*, Cm | This study |
| pYAK1-*macA_2_B_2_* | pYAK1 carrying the flanking sequence of *macA_2_B_2_*, Cm | This study |
| pMMB207 | Low-copy-number vector, Cm | Laboratory collection |
| pMMB-*macA_2_B_2_* | Plasmid pMMB207 carrying the complete *macA_2_B_2_* operon | This study |
