# Supplementary material for: The TonB system in Aeromonas hydrophila NJ-35 is essential for MacA2B2 efflux pump-mediated macrolide resistance
Source: Vet Res. 2021 Apr 29;52:63. doi: 10.1186/s13567-021-00934-w (PMC8082627; doi:10.1186/s13567-021-00934-w)
Supplement: Supplementary file 2 — Additional file 2. Primers used in this study. [file 13567_2021_934_MOESM2_ESM.doc]

**Additional file 2 Primers used in this study**

| **Primer** | **Sequence (5′–3′)** |
| --- | --- |
| Cloning |  |
| M1-P1 | CAGGTCGACTCTAGAGGATCCGAGAAGAACACCGAATCCCT |
| M1-P2 | GATATCCGCGGCACCCTCTTCGGCG |
| M1-P3 | AAGAGGGTGCCGCGGATATCCTTGGC |
| M1-P4 | GAGCTCGGTACCCGGGGATCCCTCGGTGGCGGTCAACT |
| M2-P1 | CAGGTCGACTCTAGAGGATCCCCCACAGATCCACCTCG |
| M2-P2 | CAGGTGAAACCGATGTACCACTTATCCAAACT |
| M2-P3 | TGGTACATCGGTTTCACCTGAAAGAAGCG |
| M2-P4 | GAGCTCGGTACCCGGGGATCCAAGGATTACGAGCAGGAGA |
| M2-C-F | GAGCTCGGTACCCGGGGATCCATGAACATAACTCCCCAGCA |
| M2-C-R | CAGGTCGACTCTAGAGGATCCTCATTCCCTCGCCAGC |
| qRT-PCR |  |
| *macA_2_-F* | CTCCTTCTGATCGTCCTCCTTG |
| *macA_2_-R* | GCCGTCTACTACTACGCCCTGT |
| *macB_2_-F* | GGCGGGCAGATAACCAAAC |
| *macB_2_-R* | TCGGCGTCTCCCTCTTCAT |
| *recA-F* | CGACCCCATCTATGCCGC |
| *recA-R* | CCATCTCACCTTCGATTTCCG |
