# Supplementary figures and images for: The TonB system in Aeromonas hydrophila NJ-35 is essential for MacA2B2 efflux pump-mediated macrolide resistance
Source: Vet Res. 2021 Apr 29;52:63. doi: 10.1186/s13567-021-00934-w (PMC8082627; doi:10.1186/s13567-021-00934-w)

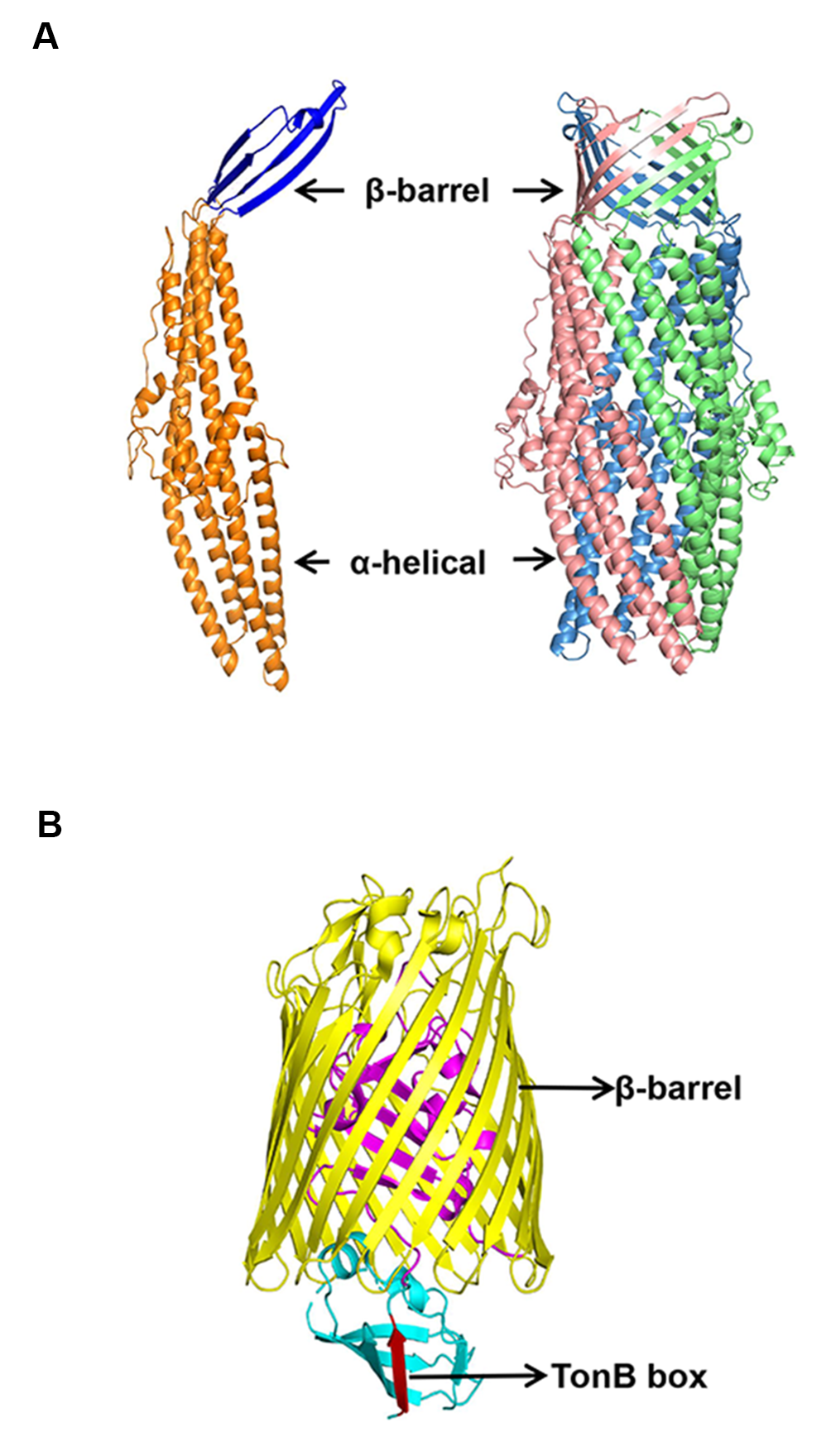

Supplement: Supplementary file 3 — Additional file 3. The 3D structure of TolC (A) and BtuB (B). The 3D structure of TolC (U876_06560) was predicted used I-TASSER online server. Three TolC monomers assemble to form a continuous conduit containing a 12-stranded β-barrel and a α-helical barrel. The monomers are individually coloured. In the single protomer, the β-barrel domain is in blue, the a-helical barrel domain is in orange. BtuB is the E. coli TonB-dependent vitamin B12 transporter. In BtuB, TonB box is in red, luminal domain is in purple and the β-barrel is in yellow. Protein Data Bank accession number 1NQH. [file 13567_2021_934_MOESM3_ESM.doc]
